# Calcium at the Helm: Mechanisms and Therapeutic Targets in the Retinal Neurovascular Unit

**DOI:** 10.3390/biom16060763

**Published:** 2026-05-22

**Authors:** Siyuan Ding, Jiayi Li, Ziyi Chen, Wen Bai, Keran Li

**Affiliations:** 1 The Fourth School of Clinical Medicine, Nanjing Medical University, Nanjing 211166, China; 2024121868@stu.njmu.edu.cn (S.D.); 2024111865@stu.njmu.edu.cn (J.L.);; 2 Department of Ophthalmology, The Affiliated Eye Hospital of Nanjing Medical University, Nanjing 210029, China

**Keywords:** calcium signaling, neurovascular unit, retina, glaucoma, diabetic retinopathy, age-related macular degeneration, TRP channels

## Abstract

Retinal neurovascular unit (RNVU) dysfunction underlies major blinding and neurodegenerative conditions including glaucoma, diabetic retinopathy (DR), age-related macular degeneration (AMD), retinal ischemia–reperfusion (RIR) injury, and Alzheimer’s disease (AD)-associated retinopathy. Within the RNVU, calcium ions coordinate neurotransmission, glial activation, vascular tone, and blood–retinal barrier maintenance, and calcium dysregulation is emerging as a unifying pathogenic hub across these conditions. Although upstream triggers differ, including mechanical stress in glaucoma, hyperglycemia in DR, oxidative damage in AMD, ischemic energy failure in RIR, and amyloid-β–driven endoplasmic reticulum stress in AD, all converge on disruption of intracellular calcium homeostasis, producing shared downstream consequences including excitotoxic injury of retinal ganglion cells (RGCs), Müller cell reactive gliosis, and pericyte hypercontraction. Broad-spectrum calcium channel blockade has shown limited clinical success, underscoring the need for cell-type-specific and pathway-selective approaches. This review therefore catalogs key interventional nodes, including transient receptor potential (TRP) channel antagonists, T-type calcium channel inhibitors, calcium/calmodulin-dependent protein kinase II (CaMKII) suppressors, and mitochondrial permeability transition pore (mPTP) inhibitors, and discusses how precision targeting of these pathways may restore RNVU homeostasis and open a therapeutic window into central nervous system (CNS) degenerative disorders.

## 1. Introduction

The retina is an extension of the central nervous system (CNS). Its function is to convert incoming light signals into neural signals for visual processing by the brain. Given that the retina consumes large amounts of energy and oxygen, its normal operation requires a precisely regulated local environment [1], and maintaining this stability relies on complex intercellular collaboration. In recent years, scientists have adopted the concept of “neurovascular unit” (NVU) from neuroscience to elucidate retinal physiology [2]. This model provides a key framework for explaining the interactions among neurons, glial cells and vascular components [3]. The main functions of the retinal neurovascular unit (RNVU) include maintaining the blood–retinal barrier (BRB), regulating local blood flow in response to neural activity through neurovascular coupling (NVC), and balancing retinal energy demand with blood supply [4,5]. Any functional impairment in any component of the unit will disrupt the stable retinal environment, leading to neurovascular dysfunction. For example, dysfunction of Müller cells leads to the accumulation of excitotoxic glutamate and disruptions in ion homeostasis, which directly impair the survival of retinal ganglion cells (RGCs) and alter local vascular tone through abnormal purinergic signaling [6].

In this precisely regulated system, calcium acts as a ubiquitous signaling molecule coordinating a variety of cellular activities [7]. At the intracellular level, calcium signals control neurotransmitter release, glial cell activation, vascular tone, and vascular permeability, as well as regulating energy metabolism, cell growth, differentiation, and apoptosis. Retinal cells maintain intracellular calcium concentration through a variety of mechanisms [8]. Under resting conditions, the concentration of calcium is extremely low (about 100 nM), but it can undergo rapid and transient increases during normal activity [9]. This strict regulation of calcium dynamics is crucial to ensure the coordinated functioning of neurons, glial cells, and vascular cells within the RNVU. Accumulating evidence indicates that calcium dysregulation is a common pathological feature of various blinding retinopathies. Therefore, to develop new therapies, it is essential to comprehensively understand the mechanism of calcium signals in the distinct cell types of the RNVU. This review discusses the normal function and regulatory mechanism of calcium in the RNVU, explores the impact of calcium homeostatic disruption on important retinal diseases, and proposes future research directions for targeted calcium-dependent neurovascular interventions.

## 2. The Concept and Importance of the Retinal Neurovascular Unit

**Figure 1:**
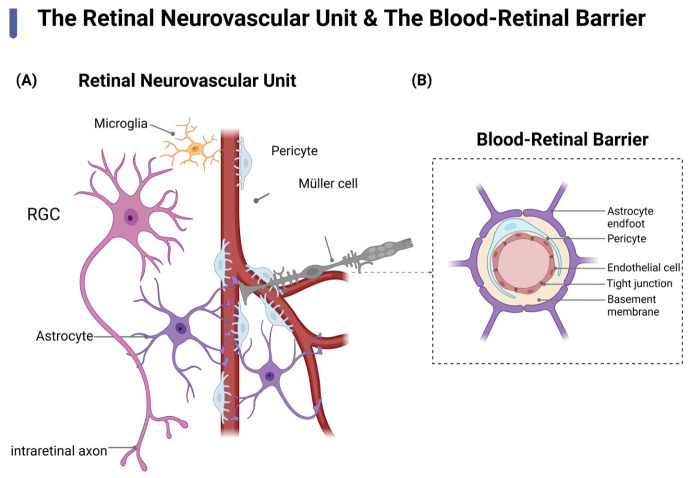
Schematic organization of the RNVU and the BRB. Created in BioRender. Ding, S. (2026) https://BioRender.com/umejmy5; accessed on 20 March 2026. (**A**) The RNVU is composed of RGCs, astrocytes, Müller glia, microglia, vascular endothelial cells, and pericytes, which interact closely to maintain retinal homeostasis, regulate neurovascular coupling, and support visual function. Beyond the inner retina, the outer retinal compartment also includes RPE cells, which form the oBRB and support photoreceptor metabolism and subretinal ionic balance. (**B**) The BRB consists of the inner blood–retinal barrier (iBRB), formed by endothelial cells, pericytes, and glial endfeet, and the oBRB, formed by RPE cells. Tight regulation of calcium homeostasis is essential for maintaining barrier integrity, vascular tone, neuronal survival, and glial function. Disruption of calcium signaling within the RNVU contributes to blood–retinal barrier breakdown, neuroinflammation, vascular dysfunction, and progressive retinal degeneration.

The RNVU is a functional network composed of neurons, Müller cells, astrocytes, microglia, endothelial cells, pericytes, smooth muscle cells, and extracellular matrix. It coordinates neurovascular coupling, metabolic support, BRB integrity, waste clearance, inflammatory regulation, and immune surveillance to maintain retinal homeostasis [2,10] (Figure 1). Beyond the inner retinal layers, the RNVU also encompasses an outer compartment in which retinal pigment epithelial (RPE) cells constitute the outer blood–retinal barrier (oBRB). As a critical component of the outer RNVU, RPE cells support photoreceptor function through phagocytosis of shed outer segments, maintain ionic and fluid homeostasis in the subretinal space, and supply essential metabolic substrates to photoreceptors [11]. Their calcium-dependent functions are therefore indispensable for outer retinal RNVU integrity and are particularly vulnerable to disruption in age-related macular degeneration (AMD) [12].

However, under pathological conditions, the interactions among these cells may be disrupted, compromising RNVU integrity. For instance, glial hyperactivation or the failure of endothelial–pericyte cooperation can disrupt NVC [13]. Endothelial–pericyte cooperation refers to the contact-dependent and paracrine crosstalk—mediated principally by PDGF-B/PDGFRβ and Angiopoietin-1/Tie2 signaling—whereby pericytes reinforce endothelial tight junctions and BRB integrity; its disruption sensitizes endothelial cells to vascular endothelial growth factor (VEGF)-driven hyperpermeability and barrier failure [14]. This uncoupling leads to barrier leakage and inflammatory responses [13,15]. These problems eventually lead to BRB breakdown, neovascularization and neurodegeneration. Increasingly, the perspective of treating RNVU impairment as a holistic functional disorder is gaining prominence. This systemic dysfunction is now recognized as a common underlying cause of various blinding diseases, including DR, glaucoma, AMD, and retinal ischemia–reperfusion injury. The RNVU is of great significance in both clinical and basic research. Clinical studies indicate that RNVU dysfunction often precedes overt microvascular lesions, serving as an early event in diseases such as DR. Therefore, early RNVU dysfunction should be interpreted not only as a vascular abnormality but also as a functional disturbance involving neurons, glia, endothelial cells, pericytes, and RPE cells. From this perspective, calcium signaling provides a mechanistic link between early cellular dysfunction and later structural damage, supporting its value as a therapeutic target within the RNVU framework.

Because the RNVU operates as an integrated network, therapeutic strategies should move beyond isolated neuronal or vascular targets and instead restore intercellular communication, BRB integrity, and glial homeostasis [16]. It is worth noting that the retina can also be used as a “window” for monitoring CNS lesions. By observing retinal changes, we can detect early signs of systemic brain diseases such as Alzheimer’s disease (AD) [17,18]. This further highlights the potential of RNVU for early diagnosis in other systemic diseases.

## 3. The Basic Regulatory Mechanisms of Intracellular Calcium Homeostasis

Intracellular calcium homeostasis depends on coordinated transmembrane transport, intracellular stores, and buffering systems. Cytosolic free calcium is maintained at ~100 nM at rest but rises rapidly and transiently upon stimulation before returning to baseline [9]. This precise spatiotemporal regulation is fundamental to cellular function. Cytosolic calcium originates from extracellular influx and intracellular stores, primarily the endoplasmic/sarcoplasmic reticulum (ER/SR) and mitochondria. A steep electrochemical gradient between extracellular (~1–2 mM) and intracellular calcium drives influx through multiple pathways, including voltage-gated calcium channels (VGCCs), receptor-operated channels, and store-operated calcium entry (SOCE) [19]. VGCCs are essential for neurotransmitter release in photoreceptors and synaptic terminals, whereas receptor-operated channels such as the transient receptor potential (TRP) family mediate responses to mechanical, osmotic, and inflammatory stimuli [8]. In non-excitable RNVU cells—such as RPE, glial, and endothelial cells—SOCE, primarily mediated by stromal interaction molecule (STIM)–Orai signaling, plays a dominant role in maintaining calcium homeostasis and is closely linked to BRB integrity [20]. In addition to the canonical STIM1–Orai1 pathway, other calcium-permeable channels may interact with Orai-dependent signaling and contribute to the fine regulation of calcium entry in a cell-type-dependent manner [21]. The relative contribution of these parallel SOCE mechanisms varies by RNVU cell type and disease state, adding complexity to therapeutic targeting of this pathway. Astrocytes ensheath retinal blood vessels via their endfeet and release vasoactive mediators in a calcium-dependent manner [22]. Upon intracellular Ca^2+^ elevation—triggered by neuronal activity or pathological stimuli—they release arachidonic acid metabolites (prostaglandins, epoxyeicosatrienoic acids), NO, and ATP, which act on adjacent smooth muscle cells and pericytes to regulate vascular tone [23,24]. This gliovascular calcium signaling mechanism forms a critical basis of NVC and is progressively impaired across multiple retinal diseases [25].

Calcium signaling mechanisms differ fundamentally between excitable and non-excitable RNVU cells. Neurons such as RGCs and photoreceptors maintain hyperpolarized resting membrane potentials (−65 to −70 mV), favoring activation of VGCCs, particularly L-type (CaV1.2) and T-type (CaV3.3) channels [20]. In contrast, non-excitable cells such as RPE cells exhibit more depolarized potentials and lack action potentials, relying predominantly on voltage-independent pathways, including TRP channels, SOCE, and ER calcium release via inositol 1,4,5-trisphosphate receptors (IP_3_R) and ryanodine receptors (RyRs) [22,23]. These cell-specific calcium entry modes are critical considerations for targeted therapeutic strategies.

Intracellular calcium storage and release are primarily governed by the ER/SR, which sequesters calcium via sarco/endoplasmic reticulum calcium ATPase (SERCA) pumps and releases it through IP_3_R and RyR channels [9]. Beyond its storage function, the ER forms specialized microdomains with the plasma membrane and mitochondria, serving as a key hub for calcium signal initiation and integration [26]. Mitochondria further modulate calcium homeostasis by taking up calcium through the mitochondrial calcium uniporter (MCU), thereby supporting metabolic processes such as the tricarboxylic acid cycle and oxidative phosphorylation [27]. However, excessive mitochondrial calcium accumulation can trigger mitochondrial permeability transition pore (mPTP) opening, reactive oxygen species (ROS) generation, and apoptosis [28]. In DR, such mitochondrial calcium overload under hyperglycemic conditions contributes to endothelial dysfunction and BRB breakdown [29]. The ER–mitochondria contact sites act as critical platforms for calcium transfer, and their structural remodeling has significant pathological implications in retinal diseases [30].

The maintenance of calcium homeostasis ultimately depends on the coordinated action of channels, pumps, exchangers, and calcium-binding proteins. Calcium channels mediate influx in response to electrical or receptor-mediated signals, while ATP-dependent pumps, including SERCA and plasma membrane calcium ATPase (PMCA) and ion exchangers, such as the sodium/calcium exchanger (NCX), restore basal calcium levels by sequestration or extrusion [31,32,33]. Calcium-binding proteins, including calmodulin (CaM), function as sensors that translate calcium signals into downstream cellular responses by modulating target proteins such as kinases and ion channels [34,35]. Disruption of this system through excessive channel activation, impaired extrusion, or reduced buffering causes calcium overload, oxidative stress, mitochondrial dysfunction, and apoptosis, thereby contributing to DR, glaucoma, and other neurodegenerative retinopathies. The major calcium channels, transporters, and calcium-binding proteins involved in RNVU calcium homeostasis are summarized in Table 1.

## 4. The Core Role of Calcium Dysregulation in Retinal Neurovascular Unit Disease

### 4.1. Glaucoma

Glaucoma is characterized by optic nerve degeneration, progressive RGC loss, and axonal damage. Although elevated IOP is the major risk factor, RNVU interactions among neurons, glia, and vascular cells further aggravate RGC degeneration and impair repair [25,43,44,45]. Calcium dyshomeostasis, particularly calcium overload, is a central driver of this process. Calcium signals regulate RGC excitability and synaptic transmission. VGCC-mediated calcium influx is required for normal RGC activity [36,46], but under glaucomatous stress, these pathways become dysregulated. TRPV4 activation under high IOP induces excessive calcium influx [47], altering action-potential properties. In parallel, calcium-activated potassium channels, including BKCa and SK channels, modulate membrane hyperpolarization and rebound excitation [48,49,50].

RGC function critically depends on the stable Ca^2+^/K^+^ balance, making these neurons highly vulnerable to glaucoma-related stress. In early IOP elevation models, RGCs exhibit a transient adaptive period of increased excitability and reduced sensitivity to extracellular K^+,^ but this response is energetically costly and short-lived [50]. Over time, calcium channel dysfunction and ion dyshomeostasis produce persistent changes in RGC excitability [36,51]. Crucially, under glaucomatous conditions, the rebound depolarization—normally a physiological event—activates pathologically upregulated T-type calcium channels, converting it into a massive calcium influx that drives intracellular calcium overload [36,52].

RGC ion-channel remodeling is strongly shaped by Müller cells, the major radial glia that regulate ion balance, metabolic support, and NVC through calcium-dependent signaling [53,54,55,56]. In glaucoma, elevated IOP activates mechanoreceptor ion channels on Müller cells—prominently TRPV4, whose synergy with TRPC1 produces dose-dependent calcium influx—while TRPV1 further amplifies calcium entry under increased hydrostatic pressure [57], and TRPC5 may contribute to RGC death via calcium overload [8,58]. Elevated IOP deforms the Müller cell membrane—particularly at endfeet contacting the vitreous—lowering the activation threshold of TRPV1, a multimodal channel sensitive to membrane stretch, protons, and lipid mediators. The resulting calcium influx persists under temperature-clamped conditions, indicating that membrane tension, rather than thermal sensitivity, is the principal activation mechanism [57]. PIEZO1/2 and TWIK-related potassium channel 1 (TREK-1) further broaden this mechanosensory network: PIEZO1/2 respond directly to membrane stretch in trabecular meshwork cells and RGCs under elevated IOP [59,60], while TREK-1 modulates resting membrane potential in Müller cells and RGCs, indirectly gating voltage-dependent calcium entry—both representing additional upstream therapeutic targets.

This mechanical stress-induced calcium disorder initiates harmful reactive gliosis, marked by GFAP upregulation, cellular hypertrophy, and morphological remodeling [6,61]. The neuropeptide Y (NPY) system—expressed in amacrine cells, RGCs, Müller cells, and microglia—normally limits calcium influx and promotes neuronal survival; in glaucoma, dysregulated NPY receptor expression and impaired downstream signaling compromise this neuroprotective role [6]. Glial activation is further associated with upregulation of intracellular calcium release channels (e.g., RyR1 co-localized with GFAP in Müller cells of DBA/2J mice), sustaining the pathological calcium signal [38]. Ion channel remodeling in Müller cells—including Kir channel downregulation and BKCa channel upregulation—likely represents a compensatory attempt to contain gliosis [62].

Reactive Müller gliosis amplifies RNVU dysfunction through impaired glutamate buffering, abnormal ATP release, and inflammatory cytokine production. Reversal or downregulation of glutamate transporters enhances RGC excitotoxicity [63]; high IOP-induced ATP release contributes to microcirculatory ischemia [56]; and TRPV4-driven TNF-α release promotes calcium-permeable AMPA receptor expression in RGCs [37,64]. These pathways converge on excitotoxicity, ischemia, neuroinflammation, and RGC death. Astrocytic calcium signaling is similarly dysregulated in glaucoma: reactive astrocytes exhibit reduced NO bioavailability and impaired prostaglandin synthesis [65,66,67], uncoupling neuronal activity from local blood flow regulation and exacerbating optic nerve head ischemia [68]. Pericytes, closely apposed to capillary endothelial cells, regulate capillary blood flow and maintain the BRB through precise intracellular calcium signaling—the basis of NVC [69]. In glaucoma, pathological stimuli such as endothelin-1 and ROS perturb pericyte calcium homeostasis, triggering sustained calcium influx that causes persistent capillary contraction and luminal narrowing, impairing retinal microcirculation autoregulation [67,69]. This neurovascular uncoupling deprives RGCs of metabolic support, inducing chronic ischemia—especially in axonal regions—and ultimately irreversible RGC apoptosis [69].

**Figure 2:**
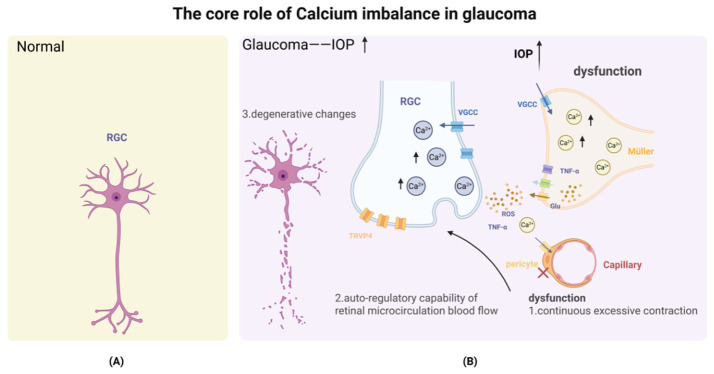
Calcium dysregulation as a central mechanism driving retinal neurovascular unit dysfunction in glaucoma; Created in BioRender. Ding, S. (2026) https://BioRender.com/swvlghi; accessed on 20 March 2026. (**A**) Elevated IOP induces pathological mechanotransduction in RGCs, Müller glia, and other RNVU components through mechanosensitive ion channels. These stimuli promote abnormal calcium influx, glial activation, altered vascular reactivity, and impaired neurovascular coupling. (**B**) Sustained calcium overload activates downstream pathogenic pathways, including mitochondrial dysfunction, endoplasmic reticulum stress, calpain activation, CaMKII signaling, inflammatory mediator release, pericyte hypercontraction, and BRB impairment, ultimately leading to RGC apoptosis, neuroinflammation, and progressive glaucomatous neurodegeneration.

Finally, BRB disruption is an important pathological feature of glaucoma, allowing toxins and immune cells to infiltrate the retina and accelerate RGC degeneration [45]. Calcium imbalance compromises the BRB through multiple mechanisms: pericyte loss undermines endothelial support, causing vascular leakage [69]; excess intracellular calcium in endothelial cells activates myosin light chain kinase, contracting the cytoskeleton and weakening tight junctions [70]; and high IOP induces mitochondrial fragmentation in retinal capillary endothelial cells, reducing claudin-5 expression and causing RGC death [71]. These mechanistic pathways are illustrated in Figure 2.

### 4.2. Diabetic Retinopathy (DR)

DR is increasingly recognized as an RNVU dysfunction disease rather than a purely microvascular complication [72]. Its pathological features include neuronal dysfunction, glial activation, pericyte loss, endothelial injury, BRB breakdown, capillary nonperfusion, edema, ischemia, and pathological neovascularization [73]. Recent studies have found that in DR, neurodegenerative changes may occur before clinically detectable vascular damage [16]. The main pathological mechanisms of DR, including oxidative stress, inflammation, neurodegeneration and vascular dysfunction, are closely related to intracellular calcium dyshomeostasis. At the same time, clinical studies show that higher serum calcium levels are independent risk factors for DR [74] and diabetic macular edema [75], highlighting the key role of calcium balance in disease development.

Hyperglycemia is the main cause of calcium imbalance in DR, which triggers ER stress and oxidative stress. As the main calcium reservoir in cells, the ER is crucial to retinal health [30]. Hyperglycemia can trigger ER stress and disrupt the signal transmission between the ER and mitochondria at the mitochondria-associated membranes (MAMs) [30,76,77]. This change promotes calcium-dependent endothelial cell apoptosis [78]. For example, the oxidative inactivation of SERCA2 at a specific site (C674) disrupts the calcium homeostasis of retinal cells (especially endothelial cells), which can lead to consequences similar to Type 1 DR [79]. Calcium imbalance also activates multiple downstream pathways, among which CaMKII is associated with increased neuronal apoptosis and mitophagy in DR under hyperglycemic conditions [42]. In other retinal cells, such as photoreceptors, calcium disorder can also activate calpains, thus exacerbating oxidative stress and inflammatory reactions [80].

**Figure 3:**
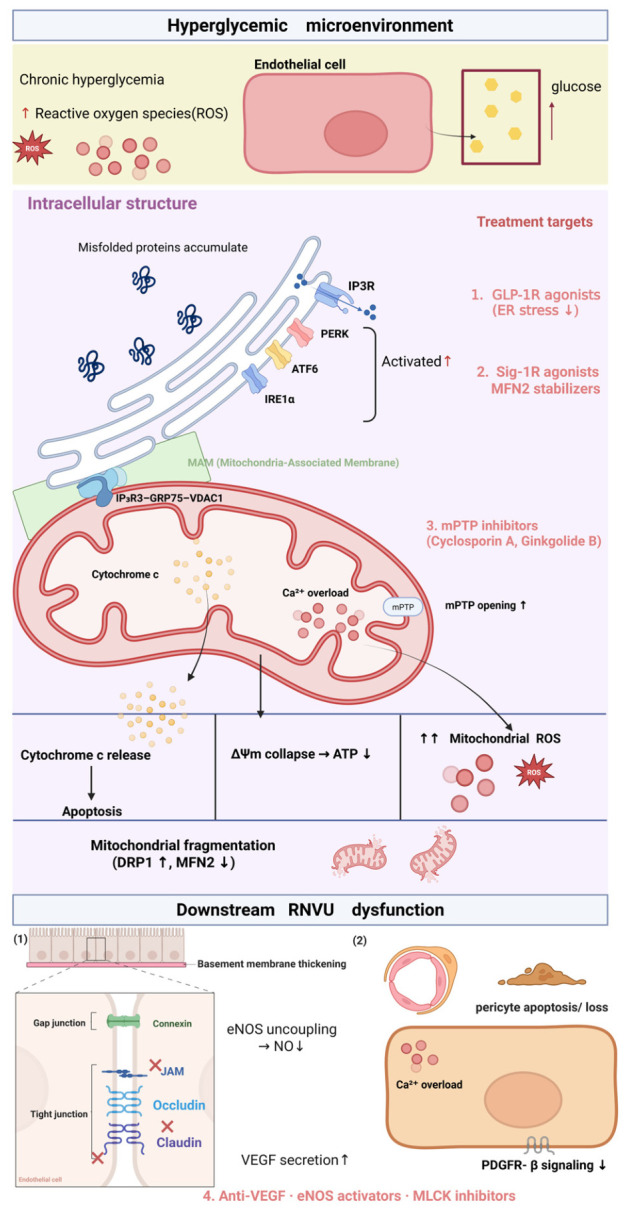
Hyperglycemia-induced calcium dysregulation and RNVU dysfunction in diabetic retinopathy. Created in BioRender. Ding, S. (2026) https://BioRender.com/d99h540; accessed on 20 March 2026. Chronic hyperglycemia and oxidative stress induce ER stress and disrupt ER–mitochondria calcium transfer through MAMs, leading to mitochondrial calcium overload, mPTP opening, mitochondrial ROS production, ATP depletion, and apoptosis. These changes contribute to endothelial injury, tight-junction disruption, eNOS uncoupling, VEGF upregulation, pericyte loss, and impaired PDGFR-β signaling, ultimately promoting BRB breakdown and RNVU dysfunction in diabetic retinopathy. Potential intervention points include GLP-1R agonists, ER stress modulators, MAM stabilizers, mPTP inhibitors, anti-VEGF therapy, eNOS activators, and MLCK inhibitors.

As early responders to hyperglycemia, Müller cells undergo significant redox and calcium homeostasis changes in DR [54]. Hyperglycemia activates the novel atypical NF-κB pro-inflammatory pathway in Müller cells through the CaMKII axis, which in turn triggers reactive gliosis, oxidative stress and mitophagy dysfunction [81]. In addition, activated Müller cells release harmful exosomes and spread inflammation and oxidative signals [82]. These processes disrupt the normal protective function of Müller cells and cause secondary damage to adjacent vascular cells—especially the pericytes and endothelial cells that form the iBRB. Dysfunction of the iBRB is a characteristic of DR, and calcium signals play a central role in this process. Hyperglycemia directly induces calcium-dependent endothelial cell apoptosis by disrupting the ER-mitochondrial coupling [78]. The normal interaction between pericytes and endothelial cells is crucial to maintaining vascular stability [83], but the imbalance of calcium caused by hyperglycemia leads to pericyte dysfunction and loss—one of the early signs of DR. These changes further lead to vascular leakage [84] and abnormal neovascularization. In fact, the integrity of the iBRB is highly dependent on the signal transmission of calcium in these cells, and the disruption of calcium regulatory functions is a key factor leading to its breakdown [20]. Calcium dysregulation can also cause neuroinflammation in DR. Hyperglycemia activates microglia [85] and induces pyroptosis through the calcium-dependent NOD-like receptor family pyrin domain containing 3 (NLRP3) inflammasome pathway [86]. Microglia- and Müller cell-driven inflammation, largely involving NF-κB signaling [87,88], further promotes RGC apoptosis, oxidative stress, and inflammatory injury under hyperglycemic conditions [89]. These mechanisms are summarized in Figure 3.

### 4.3. Age-Related Macular Degeneration (AMD)

RPE cells are highly polarized, metabolically active, and continuously exposed to light and oxidative stress. Their functions, including photoreceptor outer-segment phagocytosis, oBRB maintenance, ion-water transport, and protein clearance, depend on precise calcium signaling [11].

In dry AMD (geographic atrophy), chronic oxidative stress [30] is the main cause of RPE calcium dyshomeostasis. The core mechanism involves the dysfunction of ER–mitochondria contact sites, known as MAMs [76]. These sites facilitate bioenergetic support between the ER and mitochondria by tightly regulating calcium transfer. High oxidative stress disrupts the MAM structure and hinders the transport process [90]. This causes ER calcium leakage, mitochondrial calcium overload, bioenergetic failure, and excessive ROS generation [30,90,91]. Calcium dysregulation can activate pathways such as CaMKII [92], suppressing autophagy and mitophagy of RPE [93]. The decline in protein clearance ability [11,94] leads to the accumulation of harmful deposits (such as drusen and lipofuscin) [93,95]. Calcium imbalance, impaired autophagy and metabolic waste accumulation form a vicious cycle, which together aggravates the damage to lysosomes and mitochondria [93]. In addition, excessive calcium strongly promotes the inflammatory response of RPE. Elevated cytosolic calcium can activate the NLRP3 inflammasome [11,96], thus amplifying cell damage. Pathological calcium deposition can also form sub-RPE calcifications, marking disease progression and potentially disrupting RPE architecture [97,98].

In wet (neovascular) AMD, calcium signals are directly involved in choroidal neovascularization (CNV). In the setting of AMD, it has been proposed that TRP channel dysregulation contributes to outer BRB disruption and aberrant vessel growth [99]; however, whether TRPV4 activation ultimately promotes or restrains CNV progression remains to be fully elucidated. As sensors, these channels respond to oxidative stress and other microenvironmental signals by enhancing calcium influx and triggering angiogenesis signals. The activation of the ER/lysosomal calcium channels, such as two-pore channel 2 (TPC2), may also promote CNV and inflammation [100]. Beyond these pathways, transient receptor potential melastatin 2 (TRPM2) channels have been shown to mediate hypoxia-induced oxidative injury, inflammation, and cell death specifically in RPE cells [101], further implicating TRP channel dysregulation in outer BRB disruption during AMD progression. At the same time, calcium-related neuroinflammation driven by dysfunctional microglia or myeloid-derived cells [102] releases angiogenic factors and further promotes the formation of new blood vessels.

### 4.4. Retinal Ischemia–Reperfusion (RIR) Injury

In RIR injury, dysfunction of the RNVU is a primary driver of vision loss. The disruption of calcium homeostasis plays a pivotal role in this pathological process [40,103]. During ischemia and reperfusion, calcium overload inflicts damage at multiple levels. Mechanistically, ischemia induces energy depletion and excitotoxicity, triggering massive calcium influx. This leads to a sharp rise in intracellular calcium concentration, activating multiple damage pathways. In neurons, calcium overload is one key trigger for RGC apoptosis [104]: specifically, excessive calcium enters the mitochondria, causing the mPTP to open, which disrupts mitochondrial function and releases apoptotic factors [103]; concurrently, calcium overload induces ER stress [40], further disrupting calcium signaling and exacerbating cell damage. At the vascular level, calcium channels also contribute directly to BRB breakdown. For example, TRPV4 channels are activated in retinal vascular disease, including ischemic conditions. Increased calcium influx elevates vascular permeability, thereby promoting edema and inflammatory cell infiltration [105]. Pericytes are also damaged during RIR, further hindering microcirculation and reperfusion [106]. Notably, the body possesses protective mechanisms against calcium overload. The calcium-binding protein S100A4 can mitigate RIR damage by inhibiting ER stress and the toll-like receptor 4 (TLR4)/NF-κB inflammatory pathways [40]. This suggests that S100A4 may protect RGCs by activating the Akt signaling pathway to suppress apoptosis [41]. Based on these mechanisms, targeting calcium homeostasis and specific components of the RNVU holds therapeutic potential. Specific strategies include: using Ginkgolide B to inhibit mPTP opening [103]; regulating upstream receptors such as M1 cholinergic receptors [107], A3 adenosine receptors [108], or cannabinoid receptors [106]; and directly blocking the TRPV4 channel [105] to maintain barrier integrity.

### 4.5. Alzheimer’s Disease (AD) and Its Retinal Manifestations

The pathological link between RNVU dysfunction and CNS disease is particularly obvious in AD. AD is not limited to the brain; its retinal manifestations are increasingly regarded as a “window” into CNS pathology [109]. In the AD retina, glial components of the RNVU show marked dysfunction. Retinal microglia display altered abundance, increased ionized calcium-binding adaptor molecule 1 immunoreactivity, and abnormal three-dimensional morphology, reflecting a complex activation state that promotes chronic retinal inflammation [110,111,112]. For example, the miRNA-155/TNFSF10 signaling network has been confirmed to be related to the AD retinal inflammatory response [113]. The toxic microenvironment created by glial dysfunction and inflammatory mediators eventually leads to synaptic damage; conversely, maintaining synaptic stability has been shown to protect retinal function [114].

This neurovascular unit dysfunction is consistent with the classic “calcium hypothesis” of AD. At the cellular level, Aβ oligomers disrupt ER calcium homeostasis through at least two convergent mechanisms: sensitization of IP_3_ receptors (IP_3_R) and dysfunction of ryanodine receptor (RyR) channels, both of which increase ER calcium leak into the cytoplasm [115]. Concurrently, hyperphosphorylated tau impairs the calcium buffering capacity of mitochondria by promoting mPTP opening and reducing the ability of synaptic mitochondria to sequester excess cytosolic Ca^2+^ [116,117]. Importantly, Aβ42 has been directly localized to the ER of RGCs in post-mortem AD tissue [109], providing histological evidence that this ER calcium dysregulation operates within the retinal RNVU itself. The resulting cytosolic calcium overload activates the Ca^2+^/CaMKII and NLRP3 inflammasome pathways in Müller cells and microglia [86], amplifying the chronic neuroinflammatory state described above and further impairing Müller cell support of RGC survival [111]. While mechanistically similar calcium-dependent NLRP3 activation is observed in glaucoma [118] and DR [119], the AD retinal context is distinguished by the dual upstream drivers of Aβ-mediated ER calcium dysregulation and tau-mediated mitochondrial calcium buffering failure, highlighting calcium homeostasis regulation as a potentially disease-specific, yet convergent, therapeutic target for ocular neuroprotection. Indeed, AD-associated retinal pathology and glaucoma share key downstream calcium effector pathways: both feature microglial activation, reactive Müller cell gliosis, and TNF-α–mediated calcium-permeable AMPA receptor upregulation that together drive excitotoxic RGC death [120,121]. Although upstream triggers differ—Aβ/tau-driven ER and mitochondrial calcium dysregulation in AD versus IOP-induced mechanotransduction in glaucoma—both converge on CaMKII activation, NLRP3 inflammasome-mediated inflammation, and pericyte dysfunction [69,92,122,123]. This convergence suggests that calcium-targeting strategies explored in glaucoma models, such as TRPV4 antagonists and CaMKII inhibitors, may inform therapeutic development for AD-associated retinal pathology [64,122].

## 5. Targeted Therapeutic Strategies for Calcium Dysregulation

### 5.1. Voltage-Gated Calcium Channel-Targeted Strategies

Historically, therapeutic strategies focused on blocking VGCCs. For instance, L-type VGCC blockers such as nifedipine and verapamil [124] were shown to have dual potential by reducing calcium influx into RGCs and inducing pericyte relaxation. However, the clinical translation of such broad-spectrum blockers remains challenging. The clinical failure of the N-methyl-D-aspartate receptor (NMDAR) antagonist memantine in glaucoma [125], and the reports that amlodipine can upregulate VEGF expression in retinal cells and human serum highlight the limitations of broad-spectrum calcium interventions. Although the same study found no significant association between L-type calcium channel blocker use and severe DR risk in clinical observation [126], caution remains required. Therefore, future therapeutic directions emphasize greater selectivity. For example, in glaucoma, pathological changes in RGCs have drawn attention to T-type VGCCs, especially CaV3.3 [36], and selective T-type channel blockade may represent a promising preclinical direction. In DR, the glucagon-like peptide-1 receptor (GLP-1R) agonist exendin-4 has been shown in preclinical diabetes models to promote RGC survival by inhibiting calcium channel activity [127,128].

### 5.2. TRP Channel Antagonists as Upstream Interventions

Beyond VGCCs, emerging strategies are targeting “pathological calcium sensors” upstream of RGCs—namely, the TRP channel family, including TRPV4, TRPV1, and TRPM2. These channels convert pathological stimuli such as mechanical stress (elevated intraocular pressure) [57,64], vascular leakage-related stress [105], oxidative/hypoxic stress in RPE cells [101], and angiogenic microenvironmental cues [99,129] into harmful calcium influx. In glaucoma, selective inhibition of TRPV4 [64] or TRPV1 [57] channels expressed in Müller cells and RGCs has shown potential to suppress pressure-induced calcium toxicity at its source. In DR and RIR, TRPV4 activation contributes to endothelial calcium overload and BRB disruption [105]. In neovascular AMD, calcium signaling through TPC2 and possibly TRPV4 channels contributes to pathological CNV and vascular remodeling [100,129]. Among these, TRPV4, TRPM2, and TRPV1 are the most actionable targets. TRPV4 is linked to retinal vascular leakage, pressure-induced glial/RGC stress, and angiogenic remodeling, and its antagonist GSK2798745 has completed early-phase non-ocular clinical evaluation [130]. TRPM2 and TRPV1 provide more cell-specific targets for oxidative RPE injury in AMD [101] and pressure-induced Müller cell calcium overload in glaucoma [57], respectively.

### 5.3. Mitochondrial and Inflammatory Pathway Targeting

In addition to inhibiting calcium influx, reducing the downstream consequences of calcium overload is equally important. Excessive calcium induces mPTP opening [28] and disrupts MAMs [76,77,78], providing a rationale for drugs targeting mitochondrial calcium homeostasis. For example, mPTP inhibition with Ginkgolide B has been shown to reduce mitochondrial apoptosis in retinal ischemia–reperfusion injury [103]. Calcium signaling is also crucial in activating the NLRP3 inflammasome [86,96,131] and CaMKII [42,81,92], making NLRP3 inhibitors, CaMKII inhibitors [92], and modulators of the S100A9/TLR4 pathway [132] promising candidates for suppressing Müller cell– and microglia-mediated neuroinflammation. In AMD, calcium dysregulation may impair RPE autophagy and clearance capacity, with CaMKII signaling representing a potential upstream regulatory node [11,92,93], and targeting this pathway may restore RPE clearance capacity and reduce drusen accumulation.

### 5.4. Precision Medicine and Targeted Drug Delivery

Looking ahead, therapeutic design will likely focus on restoring integrated RNVU function through multitarget coordination and precise drug delivery. “Reprogramming” Müller cells through modulation of the NPY signaling system [6] or dopamine D1 receptor agonists that upregulate BKCa channel currents [62] can reset intracellular calcium dynamics toward a neuroprotective state. Compounds that restore calcium-dependent pericyte–endothelial communication in DR [13] or reinforce pericyte stability in ischemic retina [106] may help re-establish BRB integrity. For acute injuries such as RIR, combination therapies may offer multidimensional RNVU protection—for instance, pairing mPTP inhibitors [103] with TRPV4 antagonists [105], or combining Huperzine A-mediated cholinergic modulation [107] with pericyte-stabilizing strategies [106]. Notably, Huperzine A acts via M3 mAChR to reduce IOP and via M1 mAChR to provide retinal neuroprotection [107], making it a promising candidate within such combination regimens. At the individual level, genetic variants in calcium-activated ion channel genes—such as ANO2—have been associated with differential responses to anti-VEGF therapy in AMD [133], underscoring the need for biomarker-guided patient stratification. Emerging precision strategies may involve RNA-based therapies modulating non-coding RNAs linked to calcium-binding proteins [134,135] or engineered exosomes and lipid nanoparticles [136,137] as nanocarriers for cell-type-specific delivery of calcium-signaling modulators, including CaMKII-targeted strategies [92].

### 5.5. Limitations and Challenges

Despite the substantial progress reviewed above, several interconnected limitations continue to impede the clinical translation of calcium-targeted therapies for retinal neurovascular diseases.

The historical reliance on broad-spectrum calcium channel blockers has yielded disappointing clinical outcomes, as exemplified by the limited efficacy of L-type VGCC blockers such as nifedipine and verapamil in clinical settings [124], the failure of memantine in glaucoma trials [125], and the finding that amlodipine upregulates VEGF expression in retinal cells and human serum [126]. These findings show that non-selective calcium blockade is insufficient and may cause unintended effects, supporting more precise targeting of disease-relevant RNVU nodes. Even among supposedly selective agents, many TRP channel antagonists exhibit off-target activity at therapeutically relevant concentrations [8]—for instance, systemic TRPV4 blockade may carry dose- and tissue-dependent safety concerns, as suggested by non-ophthalmic development programs [138]—and sustained pathway inhibition risks compensatory upregulation of alternative calcium entry routes, necessitating rigorous preclinical selectivity profiling and long-term safety evaluation before clinical advancement.

Drug delivery represents an equally formidable barrier. The BRB poses significant pharmacokinetic obstacles: systemic administration frequently yields subtherapeutic RNVU concentrations, while intravitreal injection, though bypassing the BRB, is invasive and may not distribute uniformly across all retinal layers [139]. Emerging nanocarrier platforms—including engineered exosomes and lipid nanoparticles [136,137]—offer potential routes to cell-type-specific delivery, but their ocular safety, retinal penetration efficiency, and long-term tolerability require thorough investigation before clinical adoption.

Compounding these challenges, genetic polymorphisms in calcium-related genes generate substantial interindividual heterogeneity in therapeutic responses. The association between ANO2 variants and differential anti-VEGF efficacy in AMD [133] illustrates how a one-size-fits-all approach to calcium channel modulation is likely to produce inconsistent outcomes, underscoring the need for biomarker-guided patient stratification and molecular profiling in future trial designs.

Finally, much of the mechanistic evidence reviewed here derives from rodent models, which incompletely recapitulate the chronicity, genetic complexity, and cellular heterogeneity of human disease. Differences in retinal anatomy, immune microenvironment, and calcium channel expression patterns between rodents and humans further constrain direct extrapolation of preclinical findings. Bridging this translational gap will require more physiologically relevant models—such as patient-derived induced pluripotent stem cell (iPSC)-based retinal organoids, non-human primate studies, and patient-derived ex vivo preparations—alongside carefully designed early-phase clinical trials [140]. The translational stages, rationale, and limitations of these calcium-targeted strategies are summarized in Table 2.

## 6. Conclusions and Prospects

This review highlights calcium dysregulation as a central pathological axis driving RNVU dysfunction across major retinal degenerative diseases. Although upstream triggers differ—mechanical stress in glaucoma, metabolic dysregulation in DR, oxidative stress in AMD, proteotoxic stress in AD, and ischemic injury in RIR—all converge on disrupted calcium signaling within the RNVU.

Calcium overload is not merely neuron-specific; it affects multiple RNVU cell types. In RGCs, it promotes excitotoxicity and mitochondrial dysfunction; in Müller cells, it drives reactive gliosis, impaired glutamate handling, and TNF-α release; and in pericytes, it causes excessive contraction and endothelial barrier disruption. Across these compartments, calcium overload activates apoptosis-related pathways, including calpain-mediated proteolysis, mPTP-driven cytochrome c release, phospholipase A2 activation, and ER stress-induced CHOP expression [142].

In view of the limited clinical success of broad-spectrum calcium channel blockers (such as NMDAR antagonists and L-type calcium channel blockers), future therapeutic strategies must go beyond single-cell neuroprotection and precisely restore retinal neurovascular homeostasis. Promising directions include the development of selective TRPV channel antagonists, particularly TRPV4 or TRPV1 antagonists, to block upstream stress signals, targeting low-voltage-activated T-type calcium channels selectively expressed in RGCs, and inhibiting downstream pathological cascades involving CaMKII activation, NLRP3 inflammasome activation, and calpain-mediated cytoskeletal degradation. A more forward-looking strategy aims to reprogram the glial microenvironment—for example, restoring the neuroprotective phenotype of Müller cells through neuropeptide Y (NPY) agonists or BKCa channel activators. In the long run, RNA-based therapeutics, engineered exosomes, and lipid nanoparticles may help achieve cell type-specific modulation of calcium signaling within the RNVU. Engineered exosomes may be further explored for targeted delivery to RGCs or glial cells. However, their retinal penetration, targeting accuracy, long-term safety, and reproducibility require further validation before clinical translation. An in-depth understanding of calcium dynamics in the retinal neurovascular system not only reveals novel therapeutic targets for ocular diseases but also establishes an important theoretical framework for investigating degenerative diseases of the CNS, such as AD, through the unique “retinal window.”

## Figures and Tables

**Table 1:** Classification and Functions of Key Calcium Channels and Transporters in the RNVU.

Category	Key Proteins/Channels	Primary Physiological Functions in the RNVU	Pathological Role in Retinal Diseases	References
Calcium influx channels	VGCCs	Regulate RGC excitability and neurotransmitter release	Glaucoma: Upregulation of T-type channels (CaV3.3) and downregulation of L-type channels (CaV1.2) in RGCs contribute to excitotoxicity.	[36]
	TRP channels (TRPV4, TRPV1)	Act as mechanical, osmotic, and temperature sensors; involved in light adaptation and inflammatory responses	Glaucoma: TRPV4 acts as a mechanosensor; its hyperactivation in Müller cells and RGCs under elevated intraocular pressure (IOP) induces calcium influx and reactive gliosis.	[8]
	Receptor-gated channels (NMDAR)	Mediate excitatory synaptic transmission (glutamate signaling)	Glaucoma/RIR injury: Hyperactivation (excitotoxicity) leads to catastrophic calcium overload and apoptosis in RGCs	[37]
	Storage-operated calcium Entry (STIM/Orai)	Replenishes ER calcium stores; Maintains calcium homeostasis in glial and endothelial cells	DR: SOCE abnormalities are closely associated with endothelial dysfunction and BRB breakdown.	[20]
Calcium release channels	ER/SR channels (IP_3_R/RyR)	Mediate calcium release from ER stores, regulating cytoplasmic calcium kinetics	Glaucoma: Under glaucomatous conditions, RyR1 is re-localized to and induced in reactive Müller glia, co-localizing with the glial stress marker glial fibrillary acidic protein (GFAP), whereas in normal retinas RyR1 is predominantly expressed in neuronal perikarya with minimal Müller cell localization.	[38]
Calcium pumps (Active transport)	ER calcium pump (SERCA)	Pumps calcium into the ER lumen to maintain low cytoplasmic calcium concentrations	AMD/DR: SERCA dysfunction leads to ER stress, mitochondrial calcium overload, and apoptosis in RPE/endothelial cells.	[11]
	Plasma membrane calcium-ATPase (PMCA)	Actively pumps calcium out of the cell utilizing ATP	Multiple diseases: Functional impairment reduces cellular calcium clearance capacity, exacerbating cytosolic calcium overload.	[32]
Calcium exchangers (Secondary transport)	Na^+^/Ca^2+^ exchanger (NCX)	Rapidly removes intracellular calcium via the Na^+^ electrochemical gradient, especially following excitation	RIR injury: Altered expression of NCX subtypes impairs the ability of RGCs to clear calcium during the reperfusion phase.	[39]
Calcium buffers/sensors	Calcium-binding proteins (S100A4)	Bind calcium, restrict signal diffusion, and act as molecular sensors	RIR injury: S100A4 expression is dysregulated; its overexpression has been shown to protect against ER stress and inflammation.	[40,41]
	Calmodulin (CaM)	Key calcium sensor that activates calcium/calmodulin-dependent protein kinase II (CaMKII) upon calcium binding	DR: CaMKII is a key downstream effector activated under hyperglycemic conditions, driving Müller cell inflammation and RGC apoptosis.	[42]

**Table 2:** Calcium-targeted therapeutic strategies for RNVU dysfunction classified by translational stage and evidence level.

Translational Stage	Drugs/ Strategies	Primary Target or Pathway	Current Evidence and Therapeutic Rationale	Key Limitation/Note	References
Clinical/repurposing potential	GSK2798745	TRPV4 antagonist	Non-ocular human safety data; TRPV4 is linked to retinal vascular leakage.	No ocular clinical data.	[105,130]
Clinical/limited evidence	Nimodipine	L-type VGCC/vascular calcium modulation	May improve retinal microvascular perfusion in normal-tension glaucoma.	Evidence remains limited.	[141]
Clinical/negative benchmark	Memantine	NMDAR antagonist	Failed phase III glaucoma trials; illustrates limits of broad anti-excitotoxic therapy.	Use as cautionary evidence.	[125]
Preclinical/diabetes-related neuroprotection	GLP-1R agonists	GLP-1R-mediated modulation of calcium-channel activity and retinal neuroinflammation	Exendin-4 improves RGC survival in diabetic models.	Ocular indication not established.	[127]
Advanced preclinical	Selective T-type calcium channel blockers	T-type VGCCs, especially CaV3.3	T-type channel remodeling contributes to RGC injury in glaucoma models.	No ocular clinical data.	[36]
Advanced preclinical/mechanistic	TRPV4, TRPV1, and TRPM2 antagonists	Stress-sensitive TRP channels	Target pressure-, vascular-, and oxidative stress-induced calcium influx.	Selectivity and safety need validation.	[8,57,101,105]
Preclinical/downstream calcium-overload protection	Ginkgolide B	mPTP inhibition	Protects against RIR injury by reducing mitochondrial apoptosis.	Preclinical evidence only.	[103]
Preclinical/inflammatory calcium pathway	NLRP3 inhibitors; paquinimod	NLRP3 inflammasome; S100A9/TLR4 axis	Suppresses calcium-linked inflammatory injury in DR models.	Context-dependent immune effects.	[86,131,132]
Preclinical/pathway-selective	CaMKII inhibitors	Ca^2+^/CaMKII signaling	Reduces inflammation, apoptosis, and autophagy defects in retinal models.	Isoform specificity remains challenging.	[42,92]
Early stage/delivery platform	RNA-based therapies	Gene-specific modulation of calcium channels or calcium-regulatory proteins	Enables selective targeting of calcium-related genes.	Retinal delivery remains challenging.	[134,135]
Early stage / targeted delivery	Engineered exosomes and lipid nanoparticles	Cell-type-specific delivery of calcium-signaling modulators	May improve cell-targeted delivery across ocular barriers.	Long-term ocular safety unknown.	[136,137]

## Data Availability

No new data were created or analyzed in this study. Data sharing is not applicable to this article.

## Abbreviations

The following abbreviations are used in this manuscript:

AD	Alzheimer’s disease
AMD	Age-related macular degeneration
ASO	Antisense oligonucleotide
BKCa	Big-conductance calcium-activated potassium
BRB	Blood–retinal barrier
CaM	Calmodulin
CaMKII	Calcium/calmodulin-dependent protein kinase II
CNS	Central nervous system
CNV	Choroidal neovascularization
DR	Diabetic retinopathy
ER	Endoplasmic reticulum
GFAP	Glial fibrillary acidic protein
GLP-1R	Glucagon-like peptide-1 receptor
iBRB	Inner blood–retinal barrier
IOP	Intraocular pressure
IP_3_R	Inositol 1,4,5-trisphosphate receptor
MAMs	Mitochondria-associated membranes
MCU	Mitochondrial calcium uniporter
mPTP	Mitochondrial permeability transition pore
NCX	Sodium/calcium exchanger
NLRP3	NOD-like receptor family pyrin domain containing 3
NMDAR	N-methyl-D-aspartate receptor
NPY	Neuropeptide Y
NVC	Neurovascular coupling
NVU	Neurovascular unit
oBRB	Outer blood–retinal barrier
OGD/R	Oxygen-glucose deprivation/reoxygenation
PMCA	Plasma membrane calcium ATPase
RGCs	Retinal ganglion cells
RIR	Retinal ischemia–reperfusion
RNVU	Retinal neurovascular unit
ROS	Reactive oxygen species
RPE	Retinal pigment epithelium
RyR	Ryanodine receptor
SERCA	Sarco/endoplasmic reticulum calcium ATPase
SOCE	Store-operated calcium entry
STIM	Stromal interaction molecule
TLR4	Toll-like receptor 4
TNF-α	Tumor necrosis factor-alpha
TPC2	Two-pore channel 2
TREK-1	TWIK-related potassium channel 1
TRP	Transient receptor potential
TRPC	Transient receptor potential canonical
TRPM2	Transient receptor potential melastatin 2
TRPV	Transient receptor potential vanilloid
VGCCs	Voltage-gated calcium channels
VEGF	Vascular endothelial growth factor
